# Skewed macrophage polarization in aging skeletal muscle

**DOI:** 10.1111/acel.13032

**Published:** 2019-09-02

**Authors:** Chang‐Yi Cui, Riley K. Driscoll, Yulan Piao, Chee W. Chia, Myriam Gorospe, Luigi Ferrucci

**Affiliations:** ^1^ Laboratory of Genetics and Genomics, National Institute on Aging Intramural Research Program National Institutes of Health Baltimore MD USA; ^2^ Laboratory of Clinical Investigation, National Institute on Aging Intramural Research Program National Institutes of Health Baltimore MD USA; ^3^ Translational Gerontology Branch, National Institute on Aging Intramural Research Program National Institutes of Health Baltimore MD USA

**Keywords:** aging, IMAT, macrophages, polarization, skeletal muscle

## Abstract

Skeletal muscle aging is a major cause of disability and frailty in the elderly. The progressive impairment of skeletal muscle function with aging was recently linked to a disequilibrium between damage and repair. Macrophages participate in muscle tissue repair, first as pro‐inflammatory M1 subtype and then as anti‐inflammatory M2 subtype. However, information on the presence of macrophages in skeletal muscle is still sporadic and the effect of aging on macrophage phenotype remains unknown. In this study, we sought to characterize the polarization status of macrophages in skeletal muscle of persons across a wide range of ages. We found that most macrophages in human skeletal muscle are M2, and that this number increased with advancing age. On the contrary, M1 macrophages declined with aging, making the total number of macrophages invariant with older age. Notably, M2 macrophages colocalized with increasing intermuscular adipose tissue (IMAT) in aging skeletal muscle. Similarly, aged BALB/c mice showed increased IMAT and M2 macrophages in skeletal muscle, accompanied by slightly increased collagen protein production. Collectively, we report that polarization of macrophages to the major M2 subtype is associated with IMAT and propose that increased M2 in aged skeletal muscle may impact upon muscle metabolism associated with aging.

## 1 INTRODUCTION

Skeletal muscle (SKM) is the largest tissue in mammals (Janssen, 2006). Skeletal muscle comprises muscle fibers, vasculature, connective and adipose tissue, and neuromuscular junctions. The fundamental function of SKM is to provide mobility to the organism (Ferrucci et al., 2016). The progressive aging‐related decline of muscle mass and increase in connective tissue in SKM contribute to frailty, loss of mobility, and susceptibility to metabolic syndrome (Ferrucci et al., 2016; Guralnik, Ferrucci, Simonsick, Salive, & Wallace, 1995; Janssen, 2006; Kalyani, Corriere, & Ferrucci, 2014). Therefore, understanding the underlying cellular and metabolic changes in aging SKM is essential for ameliorating disabilities and diseases commonly seen in the elderly (Ferrucci et al., 2016; Kalyani et al., 2014).

Skeletal muscle aging is strongly affected by a loss of balance between damage and repair processes both at the molecular and the myocellular levels and is marked by immune activation (Gonzalez‐Freire, de Cabo, Studenski, & Ferrucci, 2014; Hepple & Rice, 2016; Kadi & Ponsot, 2010; Peake, Della Gatta, & Cameron‐Smith, 2010; Roth, Metter, Ling, & Ferrucci, 2006; Saini, McPhee, Al‐Dabbagh, Stewart, & Al‐Shanti, 2016). In particular, the balanced actions of pro‐ and anti‐inflammatory cytokines are needed to repair and remodel SKM and maintain homeostasis. These cytokines are secreted largely by polarized innate immune cells—macrophages (Arango Duque & Descoteaux, 2014). According to the classic division, polarized macrophages exist as M1, producing pro‐inflammatory cytokines, and M2, producing anti‐inflammatory cytokines and growth factors (Mills, Kincaid, Alt, Heilman, & Hill, 2000). However, the status of macrophage polarization and the function of polarized macrophages in SKM aging are poorly understood.

M2 macrophages were reported to be elevated in aged mouse SKM, associated with increased fibrosis (Wang, Wehling‐Henricks, Samengo, & Tidball, 2015). Skewed polarization of macrophages was also reported in aging human SKM, but only a few examples were examined and the results were different in each study (Kosmac et al., 2018; Przybyla et al., 2006; Tam et al., 2012). Here, we have investigated systematically M1 and M2 macrophages present in SKM biopsies from a cohort of healthy human subjects in the GESTALT (Genetic and Epigenetic Signatures of Translational Aging Laboratory Testing) study of the National Institute on Aging, ranging between 27 and 89 years of age. By fluorescent immunohistochemical analysis of SKM sections in this healthy cohort of subjects, we found M2 to be the most abundant type of macrophages in human SKM, and its numbers increased during aging. M1 macrophages were a smaller proportion of total macrophages and decreased in aged SKM. The overall consequences of these changes were that the total number of macrophages in SKM did not change with aging. Furthermore, M2 macrophages were localized in the vicinity of intermuscular adipose tissue (IMAT) in both young and aged human SKM. We also found increased IMAT in aged mice, which was accompanied by increased abundance of M2 macrophages. We further show slightly increased collagen protein deposits in aged mouse SKM, although collagen mRNA levels were significantly decreased. Our findings indicate a connection between M2 polarization and IMAT and suggest that the rise in M2 macrophages may be an adaptive response to repair aged SKM.

## 2 RESULTS

### 2.1 M2 macrophages are the most abundant subset in human SKM and their frequency increases with age

**Figure 1:**
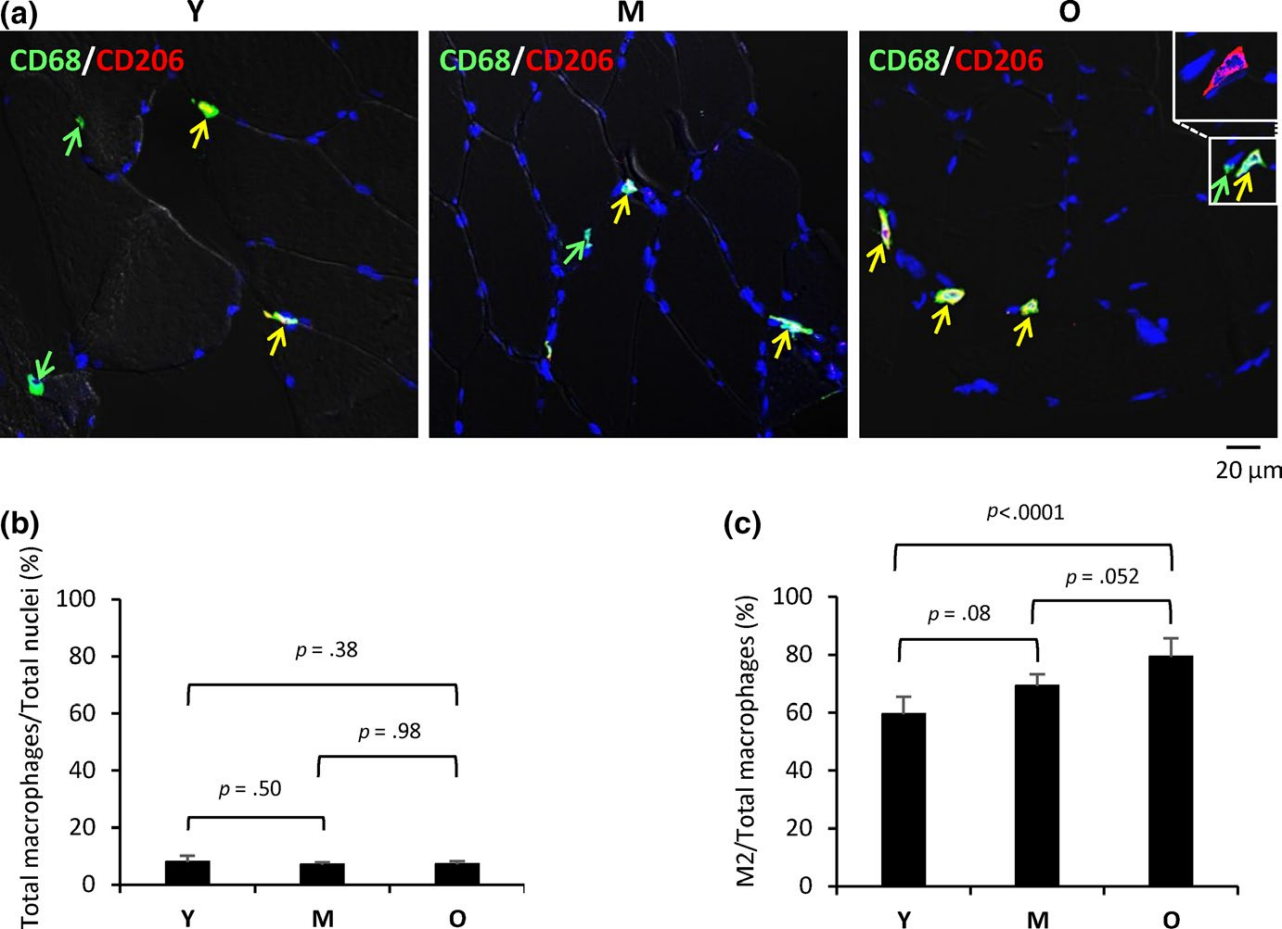
M2 macrophages increase with age in human skeletal muscle. (a) Double immunostaining of CD68 (green) and CD206 (red) to identify all macrophages and M2 macrophages, respectively, in endomysium and perimysium of skeletal muscle from young (Y), middle‐aged (M), and old (O) individuals. Inset in the upper corner (O) shows membrane localization of CD206. (b) Percent of macrophages among all cells in Y, M, and O SKM sections. (c) Quantification of M2 macrophages in SKM in each age group (% of total macrophages per field)

We investigated systematically the polarization of macrophages in human aging SKM by collecting muscle (*vastus lateralis*) biopsies from healthy young (Y), middle‐aged (M), and old (O) participants (Table 1, and see Experimental Procedures). Human muscle samples obtained using a 6‐mm Bergstrom biopsy needle were flash‐frozen, sectioned, and used for double immunostaining using antibodies that recognized the pan‐macrophage marker CD68 and the M2 marker CD206. As shown in Figure 1a, CD68+ (total) macrophages and CD68+/CD206+ (M2) macrophages were found in the perimysium (the sheath of connective tissue surrounding a bundle of muscle fibers) and endomysium (the connective tissue that surrounds each individual muscle fiber), but more macrophages were found in the perimysium in all age groups. Unlike CD68 signals, which are located in the cytosol, CD206 signals were mostly located on the cell membrane (top right inset). Pan‐macrophage signals were less than 10% of the total nuclei in all age groups, without significant differences between younger and older participants (Figure 1b). M2 macrophages comprised ~60% of all macrophage signals in the Y group (Figure 1c). Notably, M2 macrophages were significantly more abundant in M and O groups, reaching 69% and 79% of all macrophages, respectively (Figure 1c). In summary, most macrophages in noninjured healthy SKM are M2, and this population increased with aging.

**Table 1:** Human skeletal muscle samples used for this study

Y (20–39 yo)			M (40–59 yo)			O (≥60 yo)		
M2	M1	Colocalization	M2	M1	Colocalization	M2	M1	Colocalization
GT104 (35M)	GT126 (39M)	GT142 (27M)	GT060 (47M)	GT136 (55M)	GT145 (42F)	GT101 (63F)	GT132 (89M)	GT138 (71F)
GT093 (38M)	GT141 (35F)	GT141 (35F)	GT128 (49M)	GT128 (49M)	GT149 (48F)	GT105 (65F)	GT138 (71F)	GT114 (73M)
GT119 (31M)	GT119 (31M)	GT152 (38F)	GT133 (48F)	GT133 (48F)	GT162 (59M)	GT065 (73M)	GT114 (73M)	GT156 (66F)
GT122 (30M)	GT122 (30M)					GT098 (76M)	GT102 (61F)	GT159 (70M)
GT123 (28M)	GT123 (28M)					GT108 (81M)	GT159 (70M)	GT167 (73F)
GT130 (31M)	GT130 (31M)					GT111 (86M)	GT170 (79M)	
GT126 (39M)						GT114 (73M)	GT171 (61F)	
						GT102 (61F)	GT164 (81F)	
						GT159 (70M)	GT172 (88M)	
						GT170 (79M)		
						GT171 (61F)		

Note

Vastus lateralis muscle biopsies were collected from young ([Y], 20‐ to 39‐year‐old, “yo”), middle‐aged ([M], 40–59 yo], and old (≥60 yo) individuals from the GESTALT study. After sectioning, the samples were used for detection of specific cell types.

### 2.2 M1 macrophages are less abundant in human SKM and their frequency decreases with advancing age

**Figure 2:**
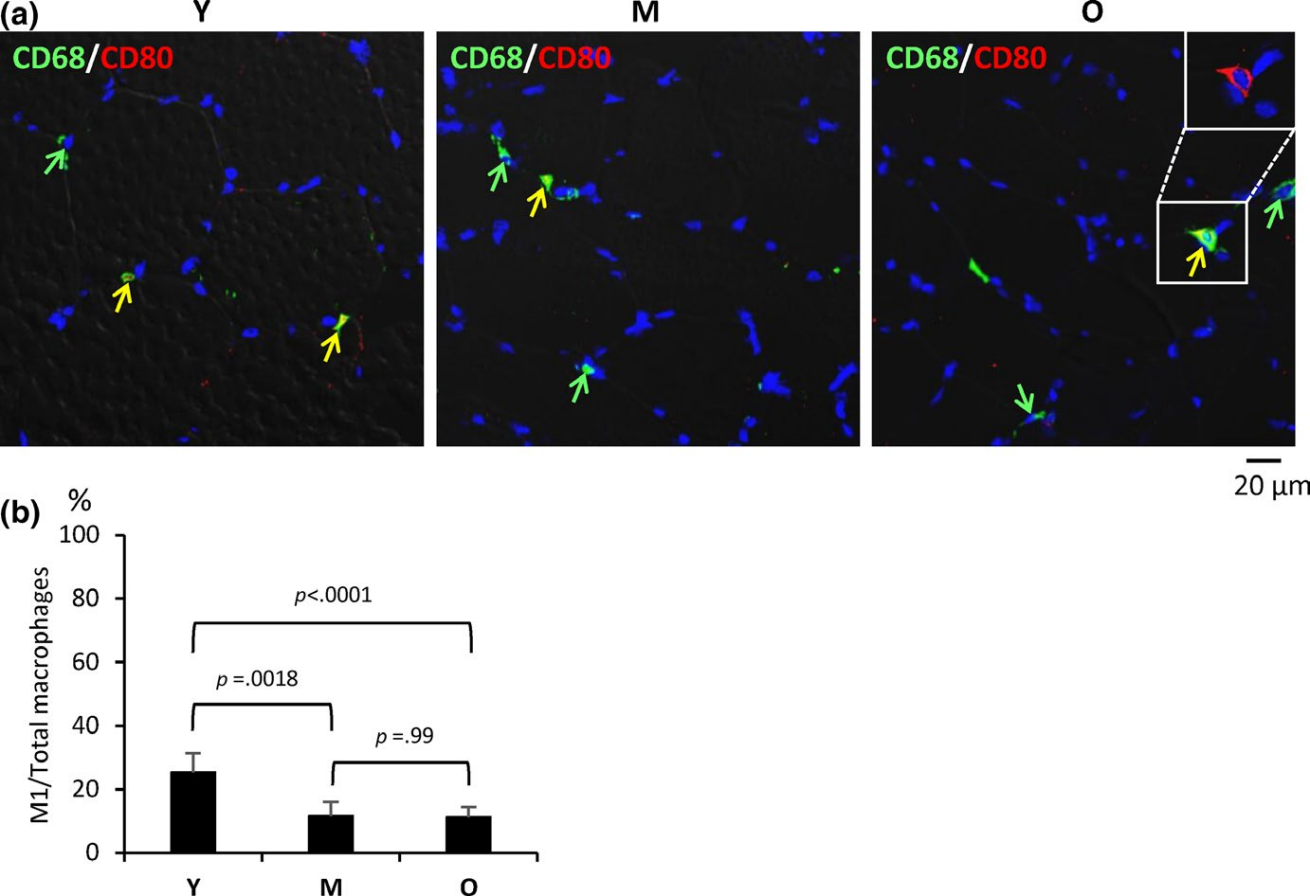
M1 macrophages decline with age in human skeletal muscle. (a) Double immunostaining of CD68 (green) and CD80 (red) to identify total macrophages and M1 macrophages, respectively, in human Y, M, and O skeletal muscle sections. In O, inset in upper corner shows membrane and cytoplasmic localization of CD80. (b) Quantification of M1 macrophages (% of total macrophages per field) in Y, M, and O skeletal muscle

The distribution of M1 macrophages in human SKM was analyzed by double immunostaining with antibodies to detect CD68 (present in all macrophages) and CD80 (present in M1 macrophages). Compared to CD68+/CD206+ macrophages (M2 type), there were substantially fewer CD68+/CD80+ macrophages (M1 type; Figure 2a). CD80 signals were found in the cytosol and/or membrane (top right inset). Quantification of the signals revealed that ~25% of all macrophages (CD68+) were M1 in the Y group, while this proportion declined significantly with aging, reaching 11% in M and O groups, respectively (Figure 2b).

### 2.3 In aging human SKM, M2 macrophages colocalize with IMAT, but not with satellite cells

**Figure 3:**
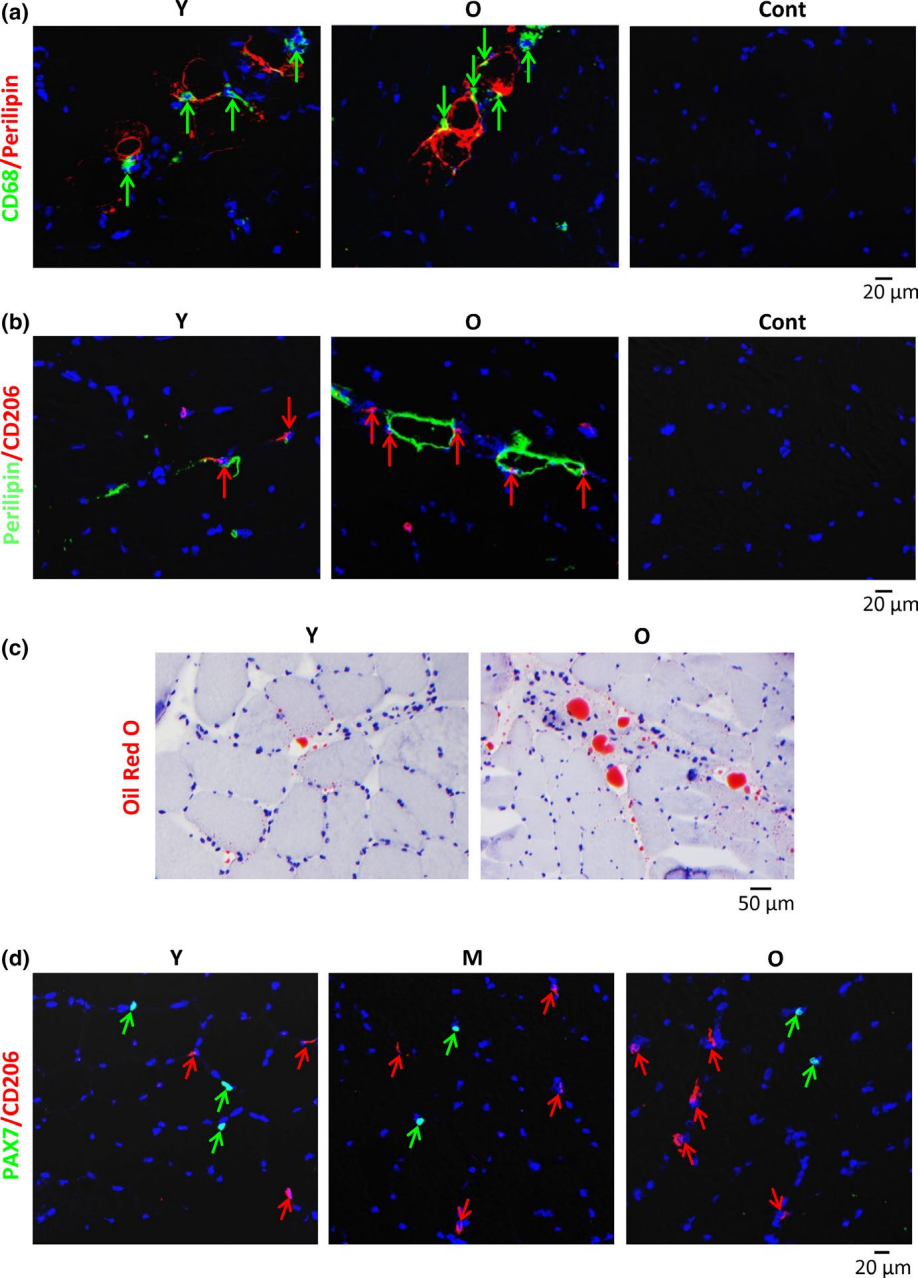
Localization of macrophages in the vicinity of adipocytes in the perimysium. (a) Double immunostaining of CD68 (green) and Perilipin 1 (red) to identify macrophages and adipocytes, respectively, in human Y and O skeletal muscle sections. (b) Relative proximity of M2 macrophages (red) and adipocytes (green) in both Y and O muscle sections. (c) Oil Red O staining of lipid droplets (red) in the perimysium. Representative sizes of droplets in O relative to Y skeletal muscle. (d) Double immunostaining of satellite cells (PAX7, green) and M2 macrophages (CD206, red) to visualize their relative localization in human Y, M, and O skeletal muscle sections

Adipocytes and exosomes released by adipose‐derived stem cells (ADSCs) have been shown to promote M2 skewing (Kang et al., 2008; Mandal, Pratt, Barnes, McMullen, & Nagy, 2011; Zhao et al., 2018). To investigate possible interactions between adipocytes and macrophages in SKM, we carried out double immunostaining to detect CD68 and the adipocyte marker perilipin 1 (PLIN1). Perilipin signals were largely restricted to the IMAT in the perimysium in both Y and O skeletal muscle (Figure 3a). Perilipin‐positive areas increased markedly in O compared to Y samples, and many CD68+ (total) macrophages were localized in the vicinity of perilipin + adipocytes (Figure 3a). Similarly, many CD206+ (M2) macrophages colocalized with adipocytes (Figure 3b). Consistent with the distribution of Perilipin, Oil Red O staining showed larger lipid drops in the perimysium of O SKM compared to Y SKM (Figure 3c). By contrast, M2 macrophages did not generally colocalize with satellite cells (muscle stem cells) in any age group, as determined by immunostaining to cells expressing CD206 and cells expressing the satellite cell marker PAX7 (Figure 3d).

### 2.4 Increased IMAT and M2 macrophages in aged mouse SKM

**Figure 4:**
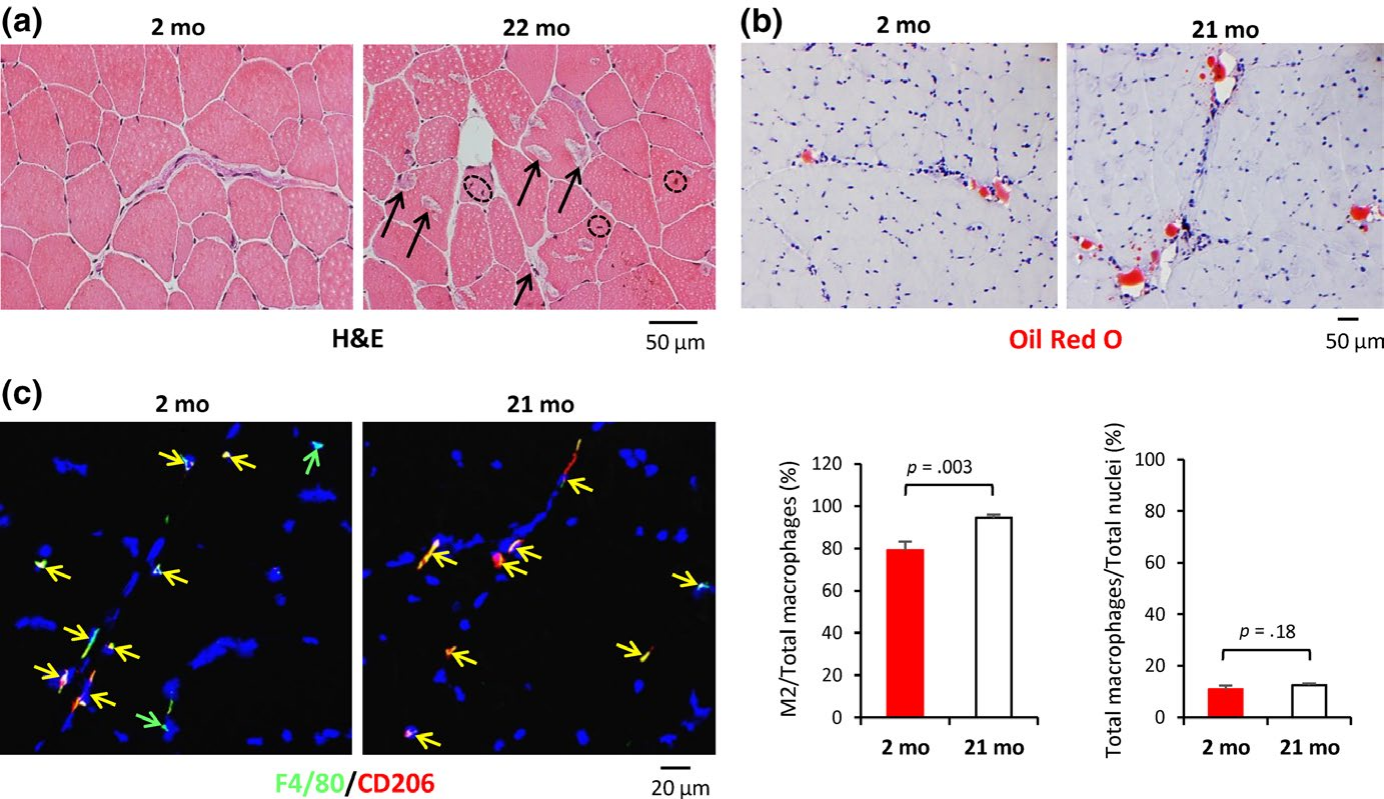
Increased intermuscular adipose tissue (IMAT) and M2 macrophages in aged skeletal muscle of BALB/c mice. (a) Histological analysis to visualize dystrophic muscle cells in old mice (arrows, right panel), but not in young mice. Notably, muscle fibers with centered nuclei were frequently observed in skeletal muscle from 22‐mo mice (broken circles) but not in 2‐mo mice. (b) Oil Red O staining was used to visualize IMAT in the perimysium of skeletal muscle (red) of 2‐ and 21‐mo mice. No obvious fat drops were observed in endomysium or intramuscle cells. (c) Left, Double immunostaining of F4/80 (green) and CD206 (red) to identify M2 macrophages in mouse SKM (left panels). Right, quantification of M2 and total macrophages in each age group

To begin to test the functional consequences of the presence of M2 as the predominant type of macrophage in human SKM and its rise with age, we analyzed aging BALB/c mice. Histology showed dystrophic muscle fibers in old (O, 21–24 months old [mo]) mice, but not in young (Y, 2 mo) mice (Figure 4a, arrows). Notably, muscle fibers with centered nuclei, a marker of regenerating cells, were frequently observed in O, but not Y mice (broken circles in Figure 4a). However, unlike the robustly regenerating myofibers, most cytoplasmic nuclei in O SKM were not located in the center of the fibers and occasionally two nuclei were found in a fiber (Figure 4a). Neither O nor Y mice showed any indication of inflammatory cell infiltration. Oil Red O staining showed increased IMAT in the perimysium area of O SKM compared to Y SKM (Figure 4b). Most macrophages in BALB/c SKM were M2; F4/80+/CD206+ M2 macrophages comprised ~79% of F4/80+ total macrophages in Y mice, which increased to ~94% in O SKM (Figure 4c**)**. Thus, as in human SKM, IMAT and M2 macrophages were increased in aged mouse SKM.

### 2.5 Adipocyte and senescent markers, as well as M2 cytokines increased in O mouse SKM

**Figure 5:**
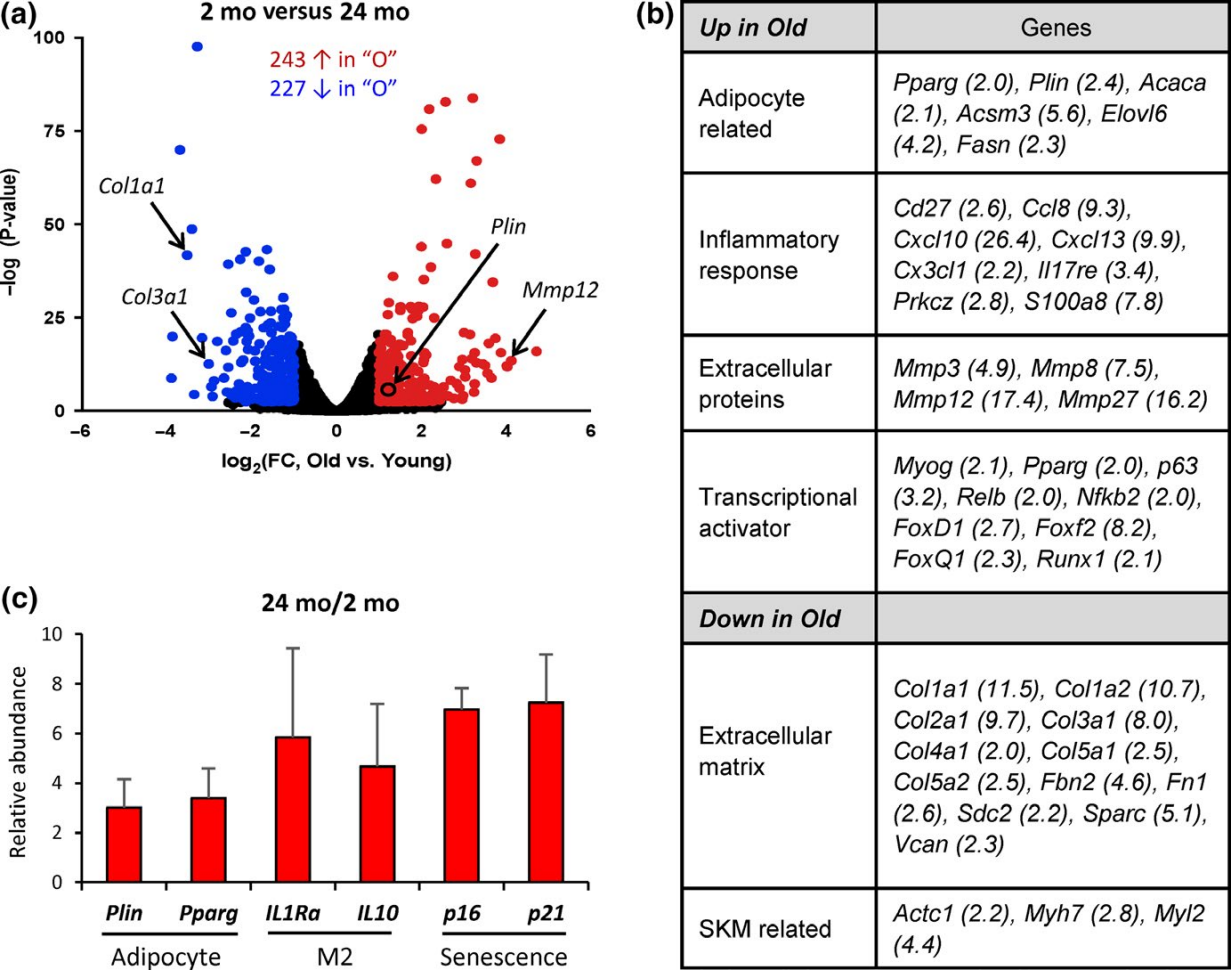
Elevated cytokines in aged mouse skeletal muscle. (a) Relative abundance of mRNAs in young (2 mo) and old (24 mo) mouse skeletal muscle, as assessed by microarray analysis. mRNAs displaying higher and lower abundance (>2‐fold, FDR < 0.05) are shown as red or blue dots, respectively. (b) GO enrichment analysis of clusters of mRNAs in muscle samples from 24‐mo versus 2‐mo mice. (c) RT‐qPCR analysis of adipocyte markers, anti‐inflammatory cytokines (M2), and senescent markers in old SKM

To understand the changes in expression patterns accompanying morphological shifts in aged SKM, we collected *vastus intermedius* muscle from Y and O mice and performed gene expression analysis using microarrays. Using twofold as the cut‐off, 243 mRNAs were more abundant, and 227 mRNAs were less abundant in O relative to Y mouse SKM (Figure 5a, Table S1; Table S2). GO enrichment analysis showed that the levels of mRNAs encoding adipocyte markers, cytokines, and transcription factors were significantly elevated in O mouse SKM (Figure 5b). By reverse transcription (RT) followed by real‐time quantitative (q)PCR analysis, we confirmed that the adipocyte markers *Plin* (*Perilipin 1*) and *Pparg* mRNAs, as well as those encoding M2 cytokines (*Il1ra* and *Il10* mRNAs), and senescent markers (*p16* and *p21* mRNAs) were significantly higher in the O group (Figure 5c).

### 2.6 The levels of *collagen* mRNA, but not collagen protein, declined in aged mouse SKM

**Figure 6:**
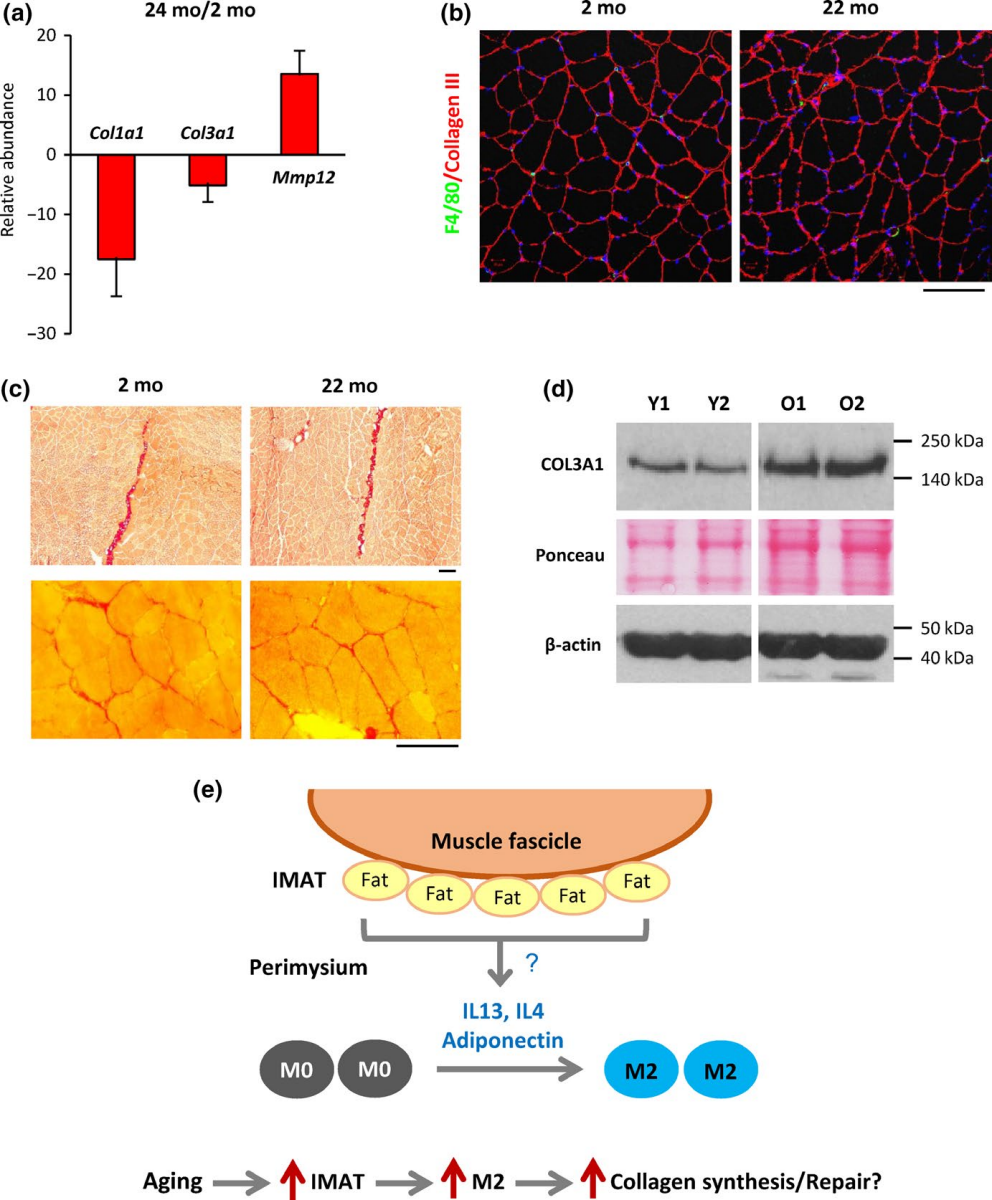
Collagen mRNA abundance, but not total collagen levels, declines in aged SKM. (a) RT‐qPCR analysis revealed markedly reduced levels of *Col1a1* and *Col3a1* mRNAs in old mouse SKM; by contrast, *Mmp12* mRNA was strongly upregulated. (b) Immunostaining showed comparable intensity of collagen III signals in young and aged mouse skeletal muscle. (c) Picrosirius red staining showed comparable collagen deposits (red) in perimysium (upper panels) and endomysium (lower panels) areas of young and old mouse SKM. (d) Western blot analysis of collagen 3 protein levels in young and old mouse SKM. (e) Model illustrating the hypothesis of macrophage polarization in SKM. Based on our results, we propose that adipocytes in the perimysium secrete cytokines that provoke M2 polarization of macrophages. In aged skeletal muscle, IMAT increases, leading to elevated cytokine secretion and a rise in M2 macrophages. Increased M2 macrophages in turn contribute to muscle tissue repair and muscle fibrosis in aged skeletal muscle likely by promoting collagen synthesis. Scale bars in (b) and (c) represent 100 µm

Finally, M2 macrophages are known to promote tissue repair in part by converting arginine to proline, which is required for collagen synthesis (Rath, Muller, Kropf, Closs, & Munder, 2014). Microarray analysis revealed that many mRNAs encoding different collagens were significantly lower in aged SKM (Figure 5a,b). By contrast, several MMPs, including MMP12, which is known to degrade collagen I and III (Taddese et al., 2010), were strikingly upregulated in O SKM (Figure 5a,b). RT‐qPCR analysis confirmed the reduced abundance of mRNAs encoding several major collagens in aged SKM, including *Col1a1* and *Col3a1* mRNAs, and upregulation of *Mmp12* mRNA (Figure 6a). However, immunostaining with specific antibodies showed comparable abundance of collagen proteins in O and Y SKM sections (Figure 6b). Picrosirius red staining, which detects collagens I and III, further showed similar collagen protein deposits both in the perimysium (Figure 6c, upper panels) and in the endomysium (Figure 6c, lower panels) in Y and O SKM. Western blot analyses using an antibody that recognized collagen III revealed slightly increased collagen levels in O SKM (Figure 6d). These data revealed that while collagen mRNA levels were significantly reduced, collagen protein abundance was normal or even slightly increased in O SKM.

## 3 DISCUSSION

Since their discovery by Metchnikoff more than a century ago (Underhill, Gordon, Imhof, Nunez, & Bousso, 2016), macrophages were shown to play diverse functions in phagocytosis, inflammation, antigen presentation, wound healing, and tumorigenesis (Shapouri‐Moghaddam et al., 2018). More recently, macrophages were found to be capable of polarizing into M1 and M2 subtypes, in turn triggering two distinct programs, pro‐inflammatory and anti‐inflammatory, respectively, that play sequential roles in the repair of tissues including SKM (Mills, 2015). Given the implication of inflammation in all aspects of age‐associated decline and disease, there is escalating interest in elucidating the role of macrophages in organ aging. Here, we have investigated the M1 and M2 macrophage subtypes in aging SKM.

### 3.1 M2 is the major type of resident macrophage in human skeletal muscle

Based on the facts that aging is characterized by a progressive loss of SKM mass and that a pro‐inflammatory state of aging has been implicated in the pathophysiology of SKM loss (Kalyani et al., 2014), we initially expected to find elevated pro‐inflammatory M1 macrophages and decreased anti‐inflammatory M2 macrophages in aged SKM (Kharraz, Guerra, Mann, Serrano, & Munoz‐Canoves, 2013; Mills, 2015). Indeed, the InCHIANTI study revealed that higher levels of the transcription factor CEBPB (CCAAT‐enhancer‐binding protein β), implicated in the shift from M1 to M2 during repair of muscle lesions, were associated with greater muscle strength and better physical performance in humans (Harries et al., 2012). This association was also seen in a mouse model bearing deletion of two CREB‐binding sites from the *CEBPB* promoter that suppressed both CEBPB induction and the transition from M1 to M2 gene expression programs, accompanying severe defects in muscle repair (Ruffell et al., 2009).

However, our data revealed just the opposite: M2 was the major type of macrophage in healthy human SKM and their presence, both in absolute numbers and in percentage of total macrophages, increased with age, while M1 macrophages showed lower abundance in human SKM and declined with age. The slightly elevated systemic inflammation seen with aging may thus be caused by additional cell types (Peake et al., 2010). Notably, M2 macrophages may also be able to secrete pro‐inflammatory cytokines as reported recently (Vogelpoel et al., 2014). Consistent with our findings, Przybyla et al. (2006) reported that M2 is the major macrophage in human SKM. These authors also showed that M1 macrophages were fewer in number and decreased in aged relative to young SKM in resting conditions (Przybyla et al., 2006). By flow cytometry, Kosmac et al. (2018) recently found that most macrophages in healthy human SKM were CD206+ (M2), but they also found that most M2 macrophages expressed the M1 marker CD86 simultaneously. This finding underscores the complex phenotypes of SKM macrophages and may also highlight the difficulty of studying M1 properties due to a current lack of robust markers. Most anti‐CD86 antibodies work poorly for immunostaining, and even the anti‐CD80 antibody used in this study provided a relatively weak signal. While new technologies, such as single‐cell sequencing analysis, can provide more accurate classification and quantification in the future, current studies support the notion that M2 macrophages are the major type of macrophage in normal human SKM. Therefore, a major function of macrophages in SKM appears to be in mediating repair, and the permanence of the pro‐inflammatory state may interfere with the subsequent restoration of muscle fibers (see below).

We also found that the number of total macrophages was unchanged between age groups, in keeping with by previous reports (Przybyla et al., 2006; Tam et al., 2012). Thus, we posit that the macrophages detected in our study are likely tissue‐resident macrophages, rather than monocytes that have migrated from the bloodstream because of injury, inflammation, or obesity (Varma et al., 2009).

### 3.2 Macrophages appear to associate closely with IMAT

Macrophage polarization is regulated by Th1 and Th2 cytokines (Mills et al., 2000). Cytokines can be released from different sources in SKM. For example, aged fibroblasts or vascular endothelial cells may release cytokines locally, and visceral adipose tissues may produce cytokines that arrive at SKM (Peake et al., 2010). Notably, adipocytes were shown to secrete IL13, IL4, and adiponectin to promote M2 polarization (Kang et al., 2008; Mandal et al., 2011), and exosomes secreted by ADSCs activate M2 polarization (Zhao et al., 2018). We found that many CD68+ macrophages located in the vicinity of adipocytes in IMAT of the perimysium in both Y and O SKM. Based on this evidence, we propose that the proximity to IMAT helps with M2 polarization of macrophages mobilized through the perimysium. Supporting this notion, many CD206+ M2 macrophages were found near IMAT in both young and aged SKM. In addition, Oil Red O staining showed that lipid droplets in the perimysium increased in aged SKM (Figure 3c) and a similar increase in IMAT was reported previously (Csapo, Malis, Sinha, Du, & Sinha, 2014). Thus, increased IMAT may contribute to increasing M2 macrophages in aged SKM, although additional cell types may also be involved in the polarization. These ideas are summarized schematically (Figure 6d).

### 3.3 Implication of M2 macrophages in SKM repair

The function of polarized macrophages in SKM injury and wound healing is well known (Kharraz et al., 2013; Rigamonti, Zordan, Sciorati, Rovere‐Querini, & Brunelli, 2014). However, the involvement of macrophages in normal SKM aging is still unclear. Histological analysis of SKM in aged BALB/c mice showed dystrophic muscle fibers morphologically resembling inclusion body myositis (IBM) (de Camargo, de Carvalho, Shinjo, de Oliveira, & Zanoteli, 2018). However, in contrast to IBM, no obvious infiltration of inflammatory cells was seen in aged BALB/c SKM, suggesting the presence of ongoing progressive muscle damage during normal aging, different from IBM. Notably, we found that the number of muscle fibers with centered nuclei increased significantly in O relative to Y SKM. However, the fibers with centered nuclei in aged SKM were much fewer in number and the distribution of the nuclei was irregular morphologically compared to the injured SKM or SKM in Duchene Muscular Dystrophy (Grounds, 2014), suggesting that attempts to repair may be less successful in aged SKM.

M2 macrophages are known to be involved in tissue repair in part by suppressing inflammation and promoting collagen synthesis (Rigamonti et al., 2014). Although it was reported that macrophages colocalize with satellite cells during wound healing (Ceafalan et al., 2018), we did not observe close localization of satellite cells with M2 macrophages in naturally aging SKM. Therefore, polarized M2 macrophages may not directly interact with muscle stem cells in healthy SKM with advancing age, although they may interact during episodes of active SKM repair, such as during reperfusion after ischemia. Elevated collagen synthesis is a major marker of tissue repair. Notably, we found that *collagen* mRNA expression was strikingly downregulated in aged SKM, consistent with previous reports (Scime et al., 2010). By contrast, collagen‐degrading MMPs were significantly more abundant in aged SKM, possibly produced by myocytes, adipocytes, or fibroblasts. However, the collagen levels in O SKM were comparable to or even slightly higher than those in Y SKM, as revealed by immunostaining, Picrosirius red staining, and Western blot analysis. These results suggest a link between increased M2 presence and higher collagen abundance, possibly due to increased collagen translation or reduced collagen degradation. In agreement with this idea, SKM macrophages were found located in the collagen‐rich perimysium and endomysium.

In closing, our data indicate that M2 macrophages increase in SKM from older individuals, suggesting that they may counteract age‐associated muscle loss. Considering that the individuals in the GESTALT cohort are exceptionally healthy, it is possible that this level of repair may not be seen in older patients who are affected by chronic medical conditions or are frail. Further studies in animal models that selectively enable or disable polarization are required to further characterize the function of macrophage subtypes in SKM aging.

## 4 EXPERIMENTAL PROCEDURES

### 4.1 Collection of human skeletal muscle biopsies

Human skeletal muscle samples were obtained from healthy young ([Y], 27‐ to 39‐year‐old [yo]), middle‐aged (M, 40–59 yo), and old (O, 60–89 yo) participants in the GESTALT (Genetic and Epigenetic Signatures of Translational Aging Laboratory Testing) study of the National Institute on Aging (NIA). The biopsies were collected at the NIA Clinical Research Branch, MedStar Harbor Hospital of Baltimore, Maryland. Vastus lateralis muscle was obtained from 34 participants (Table 1) using a 6‐mm Bergstrom biopsy needle; 10 participants for the Y group, average 33.2 yo; 7 participants for the M group, average 49.71 yo; and 17 participants for the O group, average 77.76 yo. Collected muscle samples were immediately frozen in isopentane chilled by liquid nitrogen, and then stored in −80°C until sectioning. Prior to the biopsy, the participants provided written informed consent for protocols approved by the Institutional Review Board of the NIA. All procedures were carried out in accordance with the Declaration of Helsinki.

### 4.2 Collection of SKM samples from naturally aging BALB/c mice

All animal study protocols were approved by the NIA Institutional Review Board (Animal Care and Use Committee). Young (Y, 2 month‐old, 2 mo) and old (O, 21–24 mo) inbred BALB/c mice were purchased from the NIA aged rodent colony (https://ros.nia.nih.gov/). The mice were sacrificed, and vastus intermedius muscles were taken under a dissection microscope. Collected samples were frozen in isopentane chilled by liquid nitrogen and stored in −80°C until use.

### 4.3 Immunofluorescent staining and macrophage quantification

Frozen sections were cut, fixed in cold acetone, and subjected to immunofluorescent TSA (tyramide signal amplification) staining. For double immunostaining, signals from the first primary antibody were amplified with the Tyramide SuperBoost kit (TSA AlexaFluor 488, Thermo Fisher, B40932), following the manufacturer's instructions. The signal from the second primary antibody was amplified with a biotin‐streptavidin system in which streptavidin was labeled with AlexaFluor594 as described by Kosmac et al. (2018). Primary antibodies recognizing CD68 (Abcam, ab955, 1:50 dilution), CD206 (R&D systems, AF2534, 1:200), CD80 (Abcam, ab134120, 1:50), Perilipin 1 (Cell Signaling Technology, 9349S, 1:200), Pax7 (The Developmental Studies Hybridoma Bank, 1:100), or Collagen III (Abcam, ab7778, 1:100) were used for human muscle staining. Photographs were taken using a Zeiss LSM 880 confocal microscope.

Five photographs taken by using the 20× lens from the intact part of the sections from each participant were used for macrophage quantification. Cells that stained green (CD68) and yellow (green + red; CD68 + CD206 or CD80) were counted as total macrophages; cells that stained yellow were counted as M2 macrophages (if stained for CD206) or M1 macrophages (if stained for CD80). Because of the small size of the biopsies (2 mm in diameter) and often imperfect angles of sectioning, we normalized the number of macrophages to the number of total nuclei, rather than the number of myofibers.

Frozen muscle sections from 3 young and 3 old BALB/c mice were also subjected to TSA immunostaining to quantify M2 macrophages. Primary antibodies recognizing F4/80 (Bio‐Rad, MCA497G, clone A3‐1, 1:50 dilution), the pan‐macrophage marker of mice, and CD206 (R&D systems, AF2535, 1:200), the M2 marker, were used for mouse muscle staining. Ten photographs taken by using the 20× lens from the intact part of the sections from each mouse were used for macrophage quantification.

Data were analyzed using one‐way analysis of variance (ANOVA) with Tukey's multiple comparison post‐test. A threshold of *p* value < 0.05 was used to assess the significance of observed differences. Statistical analyses were performed using graphpad prism 7.0 (GraphPad Software, Inc.).

### 4.4 RNA isolation, expression profiling, and RT‐qPCR analysis with mouse skeletal muscle

Total RNA was isolated from frozen mouse vastus intermedius muscles with Trizol (Ambion, #15596‐026) in conjunction with the PureLink RNA Mini Kit (Ambion, #12183018A) followed by on‐column Purelink DNase (Ambion, #12185‐010) treatment. 

Cyanine‐3‐labeled cRNAs were generated and hybridized to the NIA Mouse 44K Microarray v3.0 manufactured by Agilent Technologies. Three muscle samples from 3 mice in each age group were used for biological replicates. Triplicate data were analyzed by ANOVA (Cui et al., 2012). Genes with FDR < 0.05, fold difference >2.0, and mean log intensity >2.0 were considered to be significant. All data are MIAME compliant, and raw data have been deposited in GEO (GSE125481).

Reverse transcription (RT, done with SuperScript IV VILO Master Mix from Invitrogen) followed by real‐time quantitative (q)PCR analysis using Taqman probe/primer sets was performed to confirm and extend the microarray results (Applied Biosystems). RT‐qPCR analysis was used to detect *Perilipin1, PPAR‐γ, IL‐1Rα, IL‐10, p21, p16, Collagen 1a1, Collagen 3a1, and Mmp12* mRNAs*.* Each of the three sets of RNAs for each age group was assayed in triplicate. Reactions were normalized to the levels of *Gapdh* mRNA. The data were analyzed with the 2^−ΔΔCT^ method.

### 4.5 General histology and Western blot analysis of mouse SKM

The histology of young and aged mouse SKM was analyzed by staining frozen sections with Mayer's Hematoxylin solution (MHS128, Sigma) and Eosin Y solution (HT1102128, Sigma; H&E staining).

For Western blot analysis, vastus intermedius muscle was homogenized in RIPA buffer containing a protease inhibitor cocktail, PMSF, and sodium orthovanadate (Santa Cruz, SC‐24948). Homogenates were then centrifuged at 10,000 *g* for 10 min, and the supernatant was designated as Ext I. The pellets were resuspended in RIPA buffer containing 1% SDS and sonicated on ice, and the supernatant obtained by centrifugation was named Ext II (COL3A1 protein was detected in this fraction). The protein concentration of the supernatants was quantified with the Bio‐Rad Protein Assay Dye Reagent (Bradford reagent, #5000006). Protein aliquots (30 μg from each sample) were resolved on 1.0‐mm thick, 4%–12% gradient bis‐tris polyacrylamide gels (Invitrogen, NPO322PK2) and blotted onto nitrocellulose membranes (Bio‐Rad, #1704270). Membranes were blocked in 5% milk (Bio‐Rad, #1706404xtu) for 1 hr at room temperature, and all antibodies were diluted in this same solution. Membranes were incubated with primary antibodies recognizing COL3A1 (Santa Cruz, sc‐271249, 1:100) or β‐actin (Santa Cruz, sc‐47778, 1:2000) at 4°C for 16 hr. An HRP‐conjugated anti‐mouse secondary antibody (Kindle Biosciences, R1005, 1:3000‐20000) was incubated with the membrane for 45 min at 25°C. Positive bands were visualized using ECL reagent (Millipore, WBKLS0500).

### 4.6 Oil Red O staining and collagen staining

Oil Red O staining was performed using a kit from Abcam (ab150678) to analyze lipid deposits in the young and aged human and mouse SKM frozen sections, following the protocol recommended by the manufacturer. Collagens in mouse skeletal muscle were visualized with the Picrosirius Red Stain Kit (Abcam, ab150681) following the manufacturer's protocol.

## CONFLICT OF INTEREST

The authors declare no competing interests.

## AUTHORS' CONTRIBUTIONS

L.F. conceived the study; C‐Y.C. and M.G. designed experiments; C‐Y.C., R.D., and Y.P. performed experiments; C‐Y.C., M.G., and L.F. analyzed data; C.W.C, G.L., C.S., and F.I. provided technical support; and C‐Y.C., M.G., and L.F. wrote the manuscript.

## References

[acel13032-bib-0001] Arango Duque, G. , & Descoteaux, A. (2014). Macrophage cytokines: Involvement in immunity and infectious diseases. Frontiers in Immunology, 5, 491 10.3389/fimmu.2014.00491 25339958 PMC4188125

[acel13032-bib-0002] Ceafalan, L. C. , Fertig, T. E. , Popescu, A. C. , Popescu, B. O. , Hinescu, M. E. , & Gherghiceanu, M. (2018). Skeletal muscle regeneration involves macrophage‐myoblast bonding. Cell Adhesion and Migration, 12(3), 228–235. 10.1080/19336918.2017.1346774 28759306 PMC6149487

[acel13032-bib-0003] Csapo, R. , Malis, V. , Sinha, U. , Du, J. , & Sinha, S. (2014). Age‐associated differences in triceps surae muscle composition and strength – An MRI‐based cross‐sectional comparison of contractile, adipose and connective tissue. BMC Musculoskeletal Disorders, 15, 209 10.1186/1471-2474-15-209 24939372 PMC4072482

[acel13032-bib-0004] Cui, C.‐Y. , Childress, V. , Piao, Y. , Michel, M. , Johnson, A. A. , Kunisada, M. , … Schlessinger, D. (2012). Forkhead transcription factor FoxA1 regulates sweat secretion through Bestrophin 2 anion channel and Na‐K‐Cl cotransporter 1. Proceedings of the National Academy of Sciences of the United States of America, 109(4), 1199–1203. 10.1073/pnas.1117213109 22223659 PMC3268268

[acel13032-bib-0005] de Camargo, L. V. , de Carvalho, M. S. , Shinjo, S. K. , de Oliveira, A. S. B. , & Zanoteli, E. (2018). Clinical, histological, and immunohistochemical findings in inclusion body myositis. BioMed Research International, 2018, 5069042 10.1155/2018/5069042 29780824 PMC5893008

[acel13032-bib-0006] Ferrucci, L. , Cooper, R. , Shardell, M. , Simonsick, E. M. , Schrack, J. A. , & Kuh, D. (2016). Age‐related change in mobility: Perspectives from life course epidemiology and geroscience. Journals of Gerontology. Series A, Biological Sciences and Medical Sciences, 71(9), 1184–1194. 10.1093/gerona/glw043 PMC4978365 26975983

[acel13032-bib-0007] Gonzalez‐Freire, M. , de Cabo, R. , Studenski, S. A. , & Ferrucci, L. (2014). The neuromuscular junction: Aging at the crossroad between nerves and muscle. Frontiers in Aging Neuroscience, 6, 208 10.3389/fnagi.2014.00208 25157231 PMC4127816

[acel13032-bib-0008] Grounds, M. D. (2014). The need to more precisely define aspects of skeletal muscle regeneration. International Journal of Biochemistry and Cell Biology, 56, 56–65. 10.1016/j.biocel.2014.09.010 25242742

[acel13032-bib-0009] Guralnik, J. M. , Ferrucci, L. , Simonsick, E. M. , Salive, M. E. , & Wallace, R. B. (1995). Lower‐extremity function in persons over the age of 70 years as a predictor of subsequent disability. New England Journal of Medicine, 332(9), 556–561. 10.1056/NEJM199503023320902 7838189 PMC9828188

[acel13032-bib-0010] Harries, L. W. , Pilling, L. C. , Hernandez, L. D. G. , Bradley‐Smith, R. , Henley, W. , Singleton, A. B. , … Melzer, D. (2012). CCAAT‐enhancer‐binding protein‐beta expression in vivo is associated with muscle strength. Aging Cell, 11(2), 262–268. 10.1111/j.1474-9726.2011.00782.x 22152057 PMC3486692

[acel13032-bib-0011] Hepple, R. T. , & Rice, C. L. (2016). Innervation and neuromuscular control in ageing skeletal muscle. Journal of Physiology, 594(8), 1965–1978. 10.1113/JP270561 26437581 PMC4933121

[acel13032-bib-0012] Janssen, I. (2006). Influence of sarcopenia on the development of physical disability: The Cardiovascular Health Study. Journal of the American Geriatrics Society, 54(1), 56–62. 10.1111/j.1532-5415.2005.00540.x 16420198

[acel13032-bib-0013] Kadi, F. , & Ponsot, E. (2010). The biology of satellite cells and telomeres in human skeletal muscle: Effects of aging and physical activity. Scandinavian Journal of Medicine and Science in Sports, 20(1), 39–48. 10.1111/j.1600-0838.2009.00966.x 19765243

[acel13032-bib-0014] Kalyani, R. R. , Corriere, M. , & Ferrucci, L. (2014). Age‐related and disease‐related muscle loss: The effect of diabetes, obesity, and other diseases. The Lancet Diabetes and Endocrinology, 2(10), 819–829. 10.1016/S2213-8587(14)70034-8 24731660 PMC4156923

[acel13032-bib-0015] Kang, K. , Reilly, S. M. , Karabacak, V. , Gangl, M. R. , Fitzgerald, K. , Hatano, B. , & Lee, C. H. (2008). Adipocyte‐derived Th2 cytokines and myeloid PPARdelta regulate macrophage polarization and insulin sensitivity. Cell Metabolism, 7(6), 485–495. 10.1016/j.cmet.2008.04.002 18522830 PMC2586840

[acel13032-bib-0016] Kharraz, Y. , Guerra, J. , Mann, C. J. , Serrano, A. L. , & Munoz‐Canoves, P. (2013). Macrophage plasticity and the role of inflammation in skeletal muscle repair. Mediators of Inflammation, 2013, 491497 10.1155/2013/491497 23509419 PMC3572642

[acel13032-bib-0017] Kosmac, K. , Peck, B. , Walton, R. , Mula, J. , Kern, P. , Bamman, M. , … Peterson, C. (2018). Immunohistochemical identification of human skeletal muscle macrophages. BIO‐PROTOCOL, 8(12), 2883 10.21769/BioProtoc PMC6105281 30148186

[acel13032-bib-0018] Mandal, P. , Pratt, B. T. , Barnes, M. , McMullen, M. R. , & Nagy, L. E. (2011). Molecular mechanism for adiponectin‐dependent M2 macrophage polarization: Link between the metabolic and innate immune activity of full‐length adiponectin. Journal of Biological Chemistry, 286(15), 13460–13469. 10.1074/jbc.M110.204644 21357416 PMC3075692

[acel13032-bib-0019] Mills, C. D. (2015). Anatomy of a discovery: m1 and m2 macrophages. Frontiers in Immunology, 6, 212 10.3389/fimmu.2015.00212 25999950 PMC4419847

[acel13032-bib-0020] Mills, C. D. , Kincaid, K. , Alt, J. M. , Heilman, M. J. , & Hill, A. M. (2000). M‐1/M‐2 macrophages and the Th1/Th2 paradigm. The Journal of Immunology, 164(12), 6166–6173. 10.4049/jimmunol.164.12.6166 10843666

[acel13032-bib-0021] Peake, J. , Della Gatta, P. , & Cameron‐Smith, D. (2010). Aging and its effects on inflammation in skeletal muscle at rest and following exercise‐induced muscle injury. American Journal of Physiology: Regulatory, Integrative and Comparative Physiology, 298(6), R1485–R1495. 10.1152/ajpregu.00467.2009 20393160

[acel13032-bib-0022] Przybyla, B. , Gurley, C. , Harvey, J. , Bearden, E. , Kortebein, P. , Evans, W. , … Dennis, R. (2006). Aging alters macrophage properties in human skeletal muscle both at rest and in response to acute resistance exercise. Experimental Gerontology, 41(3), 320–327. 10.1016/j.exger.2005.12.007 16457979

[acel13032-bib-0023] Rath, M. , Muller, I. , Kropf, P. , Closs, E. I. , & Munder, M. (2014). Metabolism via arginase or nitric oxide synthase: Two competing arginine pathways in macrophages. Frontiers in Immunology, 5, 532 10.3389/fimmu.2014.00532 25386178 PMC4209874

[acel13032-bib-0024] Rigamonti, E. , Zordan, P. , Sciorati, C. , Rovere‐Querini, P. , & Brunelli, S. (2014). Macrophage plasticity in skeletal muscle repair. BioMed Research International, 2014, 560629 10.1155/2014/560629 24860823 PMC4016840

[acel13032-bib-0025] Roth, S. M. , Metter, E. J. , Ling, S. , & Ferrucci, L. (2006). Inflammatory factors in age‐related muscle wasting. Current Opinion in Rheumatology, 18(6), 625–630. 10.1097/01.bor.0000245722.10136.6d 17053510

[acel13032-bib-0026] Ruffell, D. , Mourkioti, F. , Gambardella, A. , Kirstetter, P. , Lopez, R. G. , Rosenthal, N. , & Nerlov, C. (2009). A CREB‐C/EBPbeta cascade induces M2 macrophage‐specific gene expression and promotes muscle injury repair. Proceedings of the National Academy of Sciences of the United States of America, 106(41), 17475–17480. 10.1073/pnas.0908641106 19805133 PMC2762675

[acel13032-bib-0027] Saini, J. , McPhee, J. S. , Al‐Dabbagh, S. , Stewart, C. E. , & Al‐Shanti, N. (2016). Regenerative function of immune system: Modulation of muscle stem cells. Ageing Research Reviews, 27, 67–76. 10.1016/j.arr.2016.03.006 27039885

[acel13032-bib-0028] Scime, A. , Desrosiers, J. , Trensz, F. , Palidwor, G. A. , Caron, A. Z. , Andrade‐Navarro, M. A. , & Grenier, G. (2010). Transcriptional profiling of skeletal muscle reveals factors that are necessary to maintain satellite cell integrity during ageing. Mechanisms of Ageing and Development, 131(1), 9–20. 10.1016/j.mad.2009.11.001 19913570

[acel13032-bib-0029] Shapouri‐Moghaddam, A. , Mohammadian, S. , Vazini, H. , Taghadosi, M. , Esmaeili, S.‐A. , Mardani, F. , … Sahebkar, A. (2018). Macrophage plasticity, polarization, and function in health and disease. Journal of Cellular Physiology, 233(9), 6425–6440. 10.1002/jcp.26429 PMC13482560 29319160

[acel13032-bib-0030] Taddese, S. , Jung, M. C. , Ihling, C. , Heinz, A. , Neubert, R. H. , & Schmelzer, C. E. (2010). MMP‐12 catalytic domain recognizes and cleaves at multiple sites in human skin collagen type I and type III. Biochimica et Biophysica Acta, 1804(4), 731–739. 10.1016/j.bbapap.2009.11.014 19932771

[acel13032-bib-0031] Tam, C. S. , Sparks, L. M. , Johannsen, D. L. , Covington, J. D. , Church, T. S. , & Ravussin, E. (2012). Low macrophage accumulation in skeletal muscle of obese type 2 diabetics and elderly subjects. Obesity (Silver Spring), 20(7), 1530–1533. 10.1038/oby.2012.24 22314623 PMC3561725

[acel13032-bib-0032] Underhill, D. M. , Gordon, S. , Imhof, B. A. , Nunez, G. , & Bousso, P. (2016). Elie Metchnikoff (1845–1916): Celebrating 100 years of cellular immunology and beyond. Nature Reviews Immunology, 16(10), 651–656. 10.1038/nri.2016.89 27477126

[acel13032-bib-0033] Varma, V. , Yao‐Borengasser, A. , Rasouli, N. , Nolen, G. T. , Phanavanh, B. , Starks, T. , … Peterson, C. A. (2009). Muscle inflammatory response and insulin resistance: Synergistic interaction between macrophages and fatty acids leads to impaired insulin action. American Journal of Physiology. Endocrinology and Metabolism, 296(6), E1300–1310. 10.1152/ajpendo.90885.2008 19336660 PMC2692398

[acel13032-bib-0034] Vogelpoel, L. T. C. , Hansen, I. S. , Rispens, T. , Muller, F. J. M. , van Capel, T. M. M. , Turina, M. C. , … den Dunnen, J. (2014). Fc gamma receptor‐TLR cross‐talk elicits pro‐inflammatory cytokine production by human M2 macrophages. Nature Communications, 5, 5444 10.1038/ncomms6444 PMC4243215 25392121

[acel13032-bib-0035] Wang, Y. , Wehling‐Henricks, M. , Samengo, G. , & Tidball, J. G. (2015). Increases of M2a macrophages and fibrosis in aging muscle are influenced by bone marrow aging and negatively regulated by muscle‐derived nitric oxide. Aging Cell, 14(4), 678–688. 10.1111/acel.12350 26009878 PMC4531081

[acel13032-bib-0036] Zhao, H. , Shang, Q. , Pan, Z. , Bai, Y. , Li, Z. , Zhang, H. , … Wang, Q. (2018). Exosomes from adipose‐derived stem cells attenuate adipose inflammation and obesity through polarizing M2 macrophages and beiging in white adipose tissue. Diabetes, 67(2), 235–247. 10.2337/db17-0356 29133512

