# Supplementary material for: Skewed macrophage polarization in aging skeletal muscle
Source: Aging Cell. 2019 Sep 2;18(6):e13032. doi: 10.1111/acel.13032 (PMC6826159; doi:10.1111/acel.13032)
Supplement: Supplementary file 1 [file ACEL-18-e13032-s001.pdf]

Table S1: Genes significantly down regulated in Old BALB/c SKM

Genes	Fold changes (O/Y)	RefSeq Acc	MGI
Spock3	0.07	NM_023689.2	MGI:1920152
Nrk	0.07		MGI:1351326
Col11a1	0.08	NM_007729.1	MGI:88446
Col1a1	0.09	NM_007742.3	MGI:88467
Col1a2	0.09	NM_007743.2	MGI:88468
Col2a1	0.1		MGI:88452
Cilp2	0.11		MGI:1915959
Col3a1	0.12	NM_009930.1	MGI:88453
1810005K13Rik	0.13		MGI:1913505
Wif1	0.14	NM_011915.1	MGI:1344332
Scg3	0.16	NM_009130.1	MGI:103032
Emid2	0.16	NM_024474.2	MGI:2155345
Rian	0.17		MGI:1922995
D0H4S114	0.17	NM_053078.3	MGI:99444
Gm266	0.18	NM_001033248.1	MGI:2685112
Has3	0.18	NM_008217.2	MGI:109599
1500015O10Rik	0.18	NM_024283.1	MGI:1926146
Lsamp	0.19		MGI:3041227
Sparc	0.19	NM_009242.1	MGI:98373
Lpar3	0.2	NM_022983.2	MGI:1929469
Fras1	0.21	NM_175473.2	MGI:2385368
Prss35	0.21	NM_178738.1	MGI:2444800
Cd247	0.21	NM_031162.1	MGI:88334
Cthrc1	0.21	NM_026778.1	MGI:1915838
C1qtnf3	0.22	NM_030888.2	MGI:1932136
Fbn2	0.22	NM_010181.2	MGI:95490
Lass1	0.22	NM_138647.1	MGI:2136690
Spsb4	0.23	NM_145134.2	MGI:2183445
Ttbk1	0.23		MGI:2147036
Ccrl1	0.23		MGI:2181676
Myl2	0.23	NM_010861.2	MGI:97272
C1qtnf6	0.23	NM_028331.2	MGI:1919959
Agtrl1	0.23	NM_011784.2	MGI:1346086
Mup4	0.23	NM_008648.1	MGI:97236
2200001I15Rik	0.23	NM_183278.1	MGI:1916384
Akap5	0.24		MGI:1918024
Mup1	0.24	NM_031188.1	MGI:97233
B830012L14Rik	0.24		MGI:2443332
Mup2	0.24	NM_001045550.1	MGI:97234
Pcdh17	0.24	NM_001013753.1	MGI:2684924
Grid2	0.24		MGI:95813
Col13a1	0.26	NM_007731.2	MGI:1277201
Atp6v0a4	0.26	NM_080467.2	MGI:2153480
Col6a3	0.26		MGI:88461
1110018H23Rik	0.28		MGI:1915759
C430049B03Rik	0.28		MGI:1924903
OTTMUSG00000007485	0.28	NM_001012323.1	MGI:3651981
1110068N23Rik	0.28		
Ptpla	0.28	NM_013935.2	MGI:1353592
Rnf182	0.28	NM_183204.1	MGI:3045355
Loxl2	0.28	NM_033325.1	MGI:2137913
Mirg	0.29		MGI:2670930
3110082D06Rik	0.29		MGI:1920485
Hpcal4	0.29	NM_174998.1	MGI:2157521
Cpz	0.3	NM_153107.2	MGI:88487
C730027P07Rik	0.3		MGI:3041196
Sfrp4	0.3	NM_016687.1	MGI:892010
Accn2	0.3	NM_009597.1	MGI:1194915
Serpinh1	0.3	NM_009825.1	MGI:88283
Chad	0.31	NM_007689.2	MGI:1096866

Fstl1	0.31	NM_008047.2	MGI:102793
Stmn4	0.31	NM_019675.2	MGI:1931224
Abi3bp	0.32		MGI:2444583
Slitrk6	0.32	NM_175499.2	MGI:2443198
Wnt2	0.32	NM_023653.3	MGI:98954
Hes5	0.32	NM_010419.2	MGI:104876
Pdgfrl	0.32	NM_026840.2	MGI:1916047
Adamts12	0.32	NM_175501.1	
A430110N23Rik	0.32	NM_173008.1	MGI:3606211
Svopl	0.33		MGI:2444335
Scube2	0.33	NM_020052.1	MGI:1928765
D430019H16Rik	0.33		MGI:2443127
Dmp1	0.33	NM_016779.2	MGI:94910
Matn4	0.33	NM_013592.2	MGI:1328314
Col6a1	0.34	NM_009933.2	MGI:88459
Abhd7	0.34	NM_001001804.1	MGI:2686228
Pdzd7	0.34	NM_177605.3	MGI:3608325
Serpinf1	0.34		MGI:108080
Egfl6	0.35	NM_019397.1	MGI:1858599
Mest	0.35	NM_008590.1	MGI:96968
Pdzd2	0.35		MGI:1922394
BC017612	0.35	NM_133214.2	MGI:3039636
Cited1	0.35	NM_007709.3	MGI:108023
Nlgn1	0.35	NM_138666.2	MGI:2179435
Thy1	0.35	NM_009382.2	MGI:98747
C130034I18Rik	0.35	NM_177233.5	MGI:2444563
E430002G05Rik	0.36	NM_173749.3	MGI:2445082
Itm2a	0.36	NM_008409.2	MGI:107706
Lrrn2	0.36	NM_010732.1	MGI:106037
Myh7	0.36	NM_080728.2	MGI:2155600
Cntnap2	0.36	NM_001004357.1	MGI:1914047
BC062109	0.36	NM_182841.1	MGI:3041258
Col12a1	0.36	NM_007730.2	MGI:88448
Pla2g12b	0.36	NM_023530.2	MGI:1917086
Pcolce	0.37		MGI:105099
Rgs9bp	0.37	NM_145840.2	MGI:2384418
Npas4	0.37	NM_153553.2	MGI:2664186
Glt8d2	0.38	NM_029102.1	MGI:1922032
EG622304	0.38		MGI:3643355
Ndrg4	0.38	NM_145602.2	MGI:2384590
Samd5	0.38	NM_177271.3	MGI:2444815
Nog	0.38	NM_008711.1	MGI:104327
Mfap5	0.38	NM_015776.2	MGI:1354387
Acan	0.38	NM_007424.2	MGI:99602
2810022L02Rik	0.39	NM_144882.2	MGI:1914448
Gm1574	0.39	NM_001005422.1	MGI:2686420
Fn1	0.39	NM_010233.1	MGI:95566
Zfp385b	0.39	NM_178723.3	MGI:2444734
Wisp1	0.39	NM_018865.2	MGI:1197008
Fgf6	0.39	NM_010204.1	MGI:95520
Pacsin1	0.39	NM_011861.1	MGI:1345181
Snap91	0.39	NM_013669.1	MGI:109132
Olfr884	0.39	NM_001011798.1	
Col5a1	0.39	NM_015734.1	MGI:88457
Nov	0.39	NM_010930.4	MGI:109185
Chst2	0.39		MGI:1891160
Col5a2	0.4	NM_007737.2	MGI:88458
Trim59	0.4	NM_025863.2	MGI:1914199
A930018M24Rik	0.4		MGI:2686053
Hmcn1	0.4		MGI:2685047
Hunk	0.4	NM_015755.2	MGI:1347352
Postn	0.4	NM_015784.2	MGI:1926321
Robo1	0.4	NM_019413.1	MGI:1274781

Col16a1	0.4	NM_028266.3	MGI:1095396
4933417E11Rik	0.41		MGI:1918384
Aldh1l2	0.41	NM_153543.1	MGI:2444680
9930023K05Rik	0.41	NM_172641.1	MGI:2443041
Olfml2b	0.41	NM_177068.3	MGI:2443310
Col24a1	0.41	NM_027770.2	MGI:1918605
D4Bwg0951e	0.41	NM_026821.2	MGI:106510
Kctd12b	0.41	NM_175429.3	MGI:2444667
C1qtnf5	0.41	NM_001040632.1	MGI:2385958
Cercam	0.41	NM_207298.2	MGI:2139134
Tub	0.41	NM_021885.4	MGI:2651573
Cdh20	0.41	NM_011800.2	MGI:1346069
Dusp10	0.42	NM_022019.2	MGI:1927070
Il17d	0.42	NM_145837.1	MGI:2446510
Xlr3c	0.42	NM_011727.1	MGI:109505
Frmd7	0.42	NM_001037750.1	MGI:2686379
Rad51ap1	0.42	NM_009013.2	MGI:1098224
Dio2	0.42	NM_010050.1	MGI:1338833
Tmc5	0.42	NM_028930.1	MGI:1921674
Tmem45a	0.42	NM_019631.2	MGI:1913122
Hist2h3c2	0.42	NM_054045.2	MGI:2448357
Grem2	0.42	NM_011825.1	MGI:1344367
Fmod	0.43	NM_021355.2	MGI:1328364
Mkx	0.43	NM_177595.2	MGI:2687286
Mfap2	0.43	NM_008546.2	MGI:99559
6430411K18Rik	0.43		
Fndc1	0.43		MGI:1915905
Tmem119	0.43	NM_146162.1	MGI:2385228
Cadm3	0.43	NM_053199.3	MGI:2137858
Vcan	0.43		MGI:102889
Loxl4	0.43	NM_053083.2	MGI:1914823
Rcn3	0.43	NM_026555.1	MGI:1277122
Bfsp1	0.43	NM_009751.2	MGI:101770
Tnmd	0.44	NM_022322.2	MGI:1929885
Col14a1	0.44	NM_181277.2	MGI:1341272
Prtg	0.44		MGI:2444710
Cd248	0.44	NM_054042.2	MGI:1917695
Sec16b	0.44	NM_033354.2	MGI:2148802
Igsf3	0.44	NM_207205.1	MGI:1926158
1500005K14Rik	0.44		MGI:1923816
Etv4	0.44	NM_008815.2	MGI:99423
Tfrc	0.44	NM_011638.3	MGI:98822
Dnmt3a	0.44	NM_007872.4	MGI:1261827
Lox	0.44	NM_010728.1	MGI:96817
Rasl10a	0.45	NM_145216.3	MGI:1922918
Pde1a	0.45	NM_016744.2	MGI:1201792
Adcy7	0.45	NM_001037724.1	MGI:102891
Ecm2	0.46		MGI:3039578
Ppp2r2b	0.46	NM_028392.1	MGI:1920180
Gpr34	0.46	NM_011823.2	MGI:1346334
Actc1	0.46	NM_009608.1	MGI:87905
Ntn4	0.46	NM_021320.2	MGI:1888978
Igsf1	0.46	NM_177591.3	MGI:2147913
Bambi	0.46	NM_026505.2	MGI:1915260
Serpinb6b	0.46	NM_011454.1	MGI:894688
Sdc2	0.46	NM_008304.2	MGI:1349165
Esm1	0.46	NM_023612.3	MGI:1918940
Insc	0.46	NM_173767.2	MGI:1917942
Lgi2	0.46	NM_144945.2	MGI:2180196
Kdelr3	0.46	NM_134090.1	MGI:2145953
Col15a1	0.47	NM_009928.2	MGI:88449
Antxr1	0.47	NM_054041.1	MGI:1916788
Hebp2	0.47	NM_019487.3	MGI:1860084

Srpr	0.47		MGI:1914648
Cd40	0.47	NM_011611.2	MGI:88336
B230217C12Rik	0.47		MGI:1915377
Rcn1	0.47	NM_009037.2	MGI:104559
BC061253	0.48		MGI:3039571
Tpbg	0.48	NM_011627.2	MGI:1341264
Cited4	0.48		MGI:1861694
Leprel2	0.48	NM_013534.4	MGI:1315208
Rsph1	0.48	NM_025290.3	MGI:1194909
Arsi	0.48		MGI:2670959
Thbs3	0.48	NM_013691.1	MGI:98739
Ptgis	0.48	NM_008968.2	MGI:1097156
Evl	0.48	NM_007965.2	MGI:1194884
Smox	0.48	NM_145533.1	MGI:2445356
1110032E23Rik	0.48	NM_133187.1	MGI:1915909
Cd33	0.48	NM_021293.1	MGI:99440
Igsf10	0.48	XM_887155.2	
Dpysl3	0.48	NM_009468.1	MGI:1349762
Etohi8	0.48		MGI:1261424
Hs6st3	0.48	NM_015820.1	MGI:1354960
Amd1	0.48	NM_009665.3	MGI:88004
2210419I08Rik	0.48		MGI:1920913
Exoc7	0.49	NM_016857.1	MGI:1859270
Mfap4	0.49	NM_029568.2	MGI:1342276
Folr2	0.49	NM_008035.1	MGI:95569
Bgn	0.49	NM_007542.3	MGI:88158
Ptn	0.49		MGI:97804
Xrcc2	0.49	NM_020570.2	MGI:1927345
Cdc6	0.49	NM_011799.2	MGI:1345150
Hmgcll1	0.49	NM_173731.2	MGI:2446108
Spin4	0.49	NM_178753.2	MGI:2444925
Col4a1	0.49	NM_009931.1	MGI:88454
Adamts2	0.49	NM_175643.2	MGI:1347356
Gpr153	0.49	NM_178406.2	MGI:1916157
Stard4	0.49		MGI:2156764
Mfrp	0.49	NM_147126.2	MGI:2385957
Ela3	0.5	NM_026419.2	MGI:1915118
Tslp	0.5	NM_021367.1	MGI:1855696
Col8a1	0.5	NM_007739.2	MGI:88463
C76566	0.5	NM_178879.3	MGI:2142841
Itgbl1	0.5	NM_145467.1	MGI:2443439
