# Supplementary material for: Skewed macrophage polarization in aging skeletal muscle
Source: Aging Cell. 2019 Sep 2;18(6):e13032. doi: 10.1111/acel.13032 (PMC6826159; doi:10.1111/acel.13032)
Supplement: Supplementary file 2 [file ACEL-18-e13032-s002.pdf]

Table S2: Genes significantly upregulated in Old BALB/c SKM

Genes	Fold changes (O/Y)	RefSeq Acc	MGI
Cxcl10	26.38	NM_021274.1	MGI:1352450
Mmp12	17.42	NM_008605.3	MGI:97005
LOC631479	16.31		
Mmp27	16.23	NM_001030289.1	MGI:3039232
Sult1e1	14.73	NM_023135.1	MGI:98431
Erc2	14.45	NM_177814.4	MGI:1098749
C130026I21Rik	13.45		MGI:3612702
Slc35f3	12.87	NM_175434.2	MGI:2444426
Gdf5	12.6	NM_008109.1	MGI:95688
Chia	12.1	NM_023186.3	MGI:1932052
Meig1	11.83	NM_008579.4	MGI:1202878
Cd163l1	11.33	NM_172909.3	MGI:2443796
Gdf6	10.91	NM_013526.1	MGI:95689
Aox3	9.89	NM_023617.2	MGI:1918974
Cxcl13	9.85	NM_018866.2	MGI:1888499
Ggt6	9.7	NM_027819.2	MGI:1918772
Mt4	9.6	NM_008631.2	MGI:99692
AI593442	9.49		MGI:2143099
Ccl8	9.29	NM_021443.1	MGI:101878
Plcxd3	9.14		MGI:2442605
2010300C02Rik	8.99		MGI:1919347
Rrad	8.86	NM_019662.2	MGI:1930943
Foxf2	8.21	NM_010225.1	MGI:1347479
Ankrd1	8.18	NM_013468.2	MGI:1097717
Cyp4f14	8.05	NM_022434.1	MGI:1927669
Clca1	7.92	NM_009899.3	MGI:1316732
S100a8	7.84	NM_013650.2	MGI:88244
Ces2	7.82	NM_145603.1	MGI:2385905
Mmp8	7.47	NM_008611.2	MGI:1202395
Dpp10	6.84	NM_199021.2	MGI:2442409
Ostn	6.48	NM_198112.1	MGI:2677164
1700027A23Rik	6.08	NM_029604.1	MGI:1923670
Pak1	5.95	NM_011035.2	MGI:1339975
4930558C23Rik	5.77		MGI:1914904
Acsm3	5.57	NM_016870.3	MGI:99538
Oit1	5.32	NM_146050.2	MGI:1201784
Clca3	5.24	NM_017474.1	MGI:1346342
Amy2-2	5.08	NM_001042711.1	MGI:104547
Eda2r	4.94		MGI:2442860
Mmp3	4.92	NM_010809.1	MGI:97010
Ankrd55	4.84		MGI:1924568
Eraf	4.84	NM_133245.1	MGI:2158492
2310039D24Rik	4.81		MGI:1922954
C4a	4.69	NM_011413.1	MGI:98320
Alb	4.64	NM_009654.1	MGI:87991
Pkp2	4.62	NM_026163.1	MGI:1914701
Rhpn2	4.55	NM_027897.2	MGI:1289234
Fgf21	4.34	NM_020013.2	MGI:1861377
Orm1	4.28	NM_008768.1	MGI:97443
Slc23a3	4.21	NM_194333.2	MGI:104516
Ctxn3	4.21		
Tead4	4.19		MGI:106907
9830102E05Rik	4.19	NM_177787.3	MGI:3607714
Elovl6	4.18	NM_130450.2	MGI:2156528
Tmem45b	4.18	NM_144936.1	MGI:2384574
Cpne2	4.18	NM_153507.2	MGI:2387578
6530401D17Rik	4.15	NM_029541.1	MGI:1923469
Cd209a	4.14	NM_133238.2	MGI:2157942
Tdrd6	4.12	NM_198418.1	MGI:2679727
Mib1	4.12	NM_144860.2	MGI:2443157

D9Ertd280e	4.04	NM_177775.3	MGI:1098699
Amy1	4.03	NM_007446.1	MGI:88019
Cd300lf	4.02	NM_145634.2	MGI:2442359
C4b	4.01	NM_009780.1	MGI:88228
Kcnj10	4	NM_001039484.1	MGI:1194504
Kcne1l	4	NM_021487.1	MGI:1913490
E030003E18Rik	4		MGI:2443348
Mt2	3.86	NM_008630.1	MGI:97172
Exph5	3.85		MGI:2443248
Mogat1	3.82	NM_026713.1	MGI:1915643
Cpeb2	3.8	NM_175937.2	MGI:2442640
Cdkn1a	3.8	NM_007669.2	MGI:104556
Rasd2	3.73		MGI:1922391
AU018778	3.71	NM_144930.1	MGI:2142687
Scd1	3.63	NM_009127.3	MGI:98239
9530080O11Rik	3.6	NM_175680.2	MGI:2441751
Kng1	3.52	NM_023125.2	MGI:1097705
Tubb6	3.52	NM_026473.2	MGI:1915201
Gsta3	3.48	NM_001077353.1	MGI:95856
Inmt	3.47	NM_009349.1	MGI:102963
EG624219	3.47		MGI:3643534
2810032G03Rik	3.46	NM_028318.2	MGI:1919919
Serpina3n	3.4	NM_009252.1	MGI:105045
A730060N03Rik	3.4		MGI:2444190
2900062L11Rik	3.4	NM_029823.1	MGI:1924226
1810022C23Rik	3.39	NM_026947.3	MGI:1916373
Il17re	3.38	NM_145826.2	MGI:1889371
4931408D14Rik	3.36		MGI:1924309
Grin3b	3.36	NM_130455.2	MGI:2150393
A2m	3.32	NM_175628.2	MGI:2449119
Cds1	3.28	NM_173370.3	MGI:1921846
Acvr1c	3.26	NM_001033369.1	MGI:2661081
Fgf10	3.26	NM_008002.3	MGI:1099809
Shroom3	3.22	NM_015756.2	MGI:1351655
Slc1a1	3.21	NM_009199.2	MGI:105083
Cd209d	3.2	NM_130904.2	MGI:2157947
Trp63	3.2	NM_011641.1	MGI:1330810
Nap1l5	3.15	NM_021432.1	MGI:1923555
Aldh1a7	3.1	NM_011921.2	MGI:1347050
E130218I03Rik	3.04		MGI:3528958
Snf1lk	3.04	NM_010831.2	MGI:104754
Gadd45a	3.04	NM_007836.1	MGI:107799
Gsta1	3.03	NM_008181.2	MGI:1095417
Tiam2	2.98	NM_011878.1	MGI:1344338
Actr3b	2.97		MGI:2661120
EG653016	2.97	XM_001000918.1	
Brsk2	2.96	NM_029426.2	MGI:1923020
4922505G16Rik	2.95	NM_001039557.1	MGI:3603828
Gm1305	2.91		MGI:2686151
Tmem8	2.85	NM_021793.1	MGI:1926283
Prkcz	2.84	NM_008860.2	MGI:97602
Katnal2	2.81		MGI:1924234
BC033932	2.8		MGI:3525074
AK129341	2.78	NM_001045524.1	MGI:2680221
Il12rb1	2.78	NM_008353.1	MGI:104579
Gdf7	2.77	NM_013527.1	MGI:95690
Gsdma1	2.77	NM_021347.2	MGI:1889509
Gm1157	2.75	NM_001033473.1	
Arrdc2	2.74	NM_027560.1	MGI:1918057
Slc25a1	2.73	NM_153150.1	MGI:1345283
Serpina1a	2.73	NM_009243.3	MGI:891971
Dntt	2.72	NM_009345.2	MGI:98659
Tdrkh	2.7		MGI:1919884

Nmbr	2.69	NM_008703.2	MGI:1100525
Apoc1	2.69	NM_007469.2	MGI:88053
Ccdc85a	2.69	NM_181577.2	MGI:2445069
4833427F10Rik	2.69		MGI:1921851
Prkar2b	2.68	NM_011158.3	MGI:97760
Foxd1	2.67	NM_008242.1	MGI:1347463
Tmco4	2.66	NM_029857.2	MGI:1924306
Ttc9	2.65	NM_001033149.1	MGI:1916730
AI851790	2.64	NM_182807.2	MGI:2143691
Ttc25	2.63		MGI:1921657
1810032O08Rik	2.6		MGI:1913543
Tmem100	2.59	NM_026433.1	MGI:1915138
Cd27	2.59	NM_001033126.2	MGI:88326
Itgax	2.58	NM_021334.2	MGI:96609
Itgb8	2.57		MGI:1338035
LOC674832	2.54	XM_977358.1	
4430402I18Rik	2.53	NM_198651.2	MGI:1918036
Slc6a7	2.52	NM_201353.1	MGI:2147363
Snca	2.52	NM_001042451.1	MGI:1277151
Ctsw	2.52	NM_009985.2	MGI:1338045
Rai14	2.52	NM_030690.2	MGI:1922896
Ripply1	2.52	NM_001037915.1	MGI:3614797
Itih3	2.51	NM_008407.1	MGI:96620
Tnfrsf12a	2.49	NM_013749.1	MGI:1351484
Krt18	2.49	NM_010664.1	MGI:96692
2310040C09Rik	2.48	NM_178618.3	MGI:1916890
2610019F03Rik	2.47	NM_173744.2	MGI:1919398
1600029D21Rik	2.47	NM_029639.1	MGI:1923759
Polq	2.46	NM_029977.1	MGI:2155399
LOC548103	2.44		
OTTMUSG00000001246	2.43	NM_001013393.1	
Fetub	2.43		MGI:1890221
2010107G23Rik	2.43	NM_027251.2	MGI:1917144
A630034I12Rik	2.42		MGI:3041215
Upp2	2.42		MGI:1923904
Cpne7	2.42	NM_170684.2	MGI:2142747
Hsf2bp	2.41		MGI:1921627
1300007F04Rik	2.39	NM_026185.2	MGI:1914727
Atp1a3	2.39	NM_144921.1	MGI:88107
Xrra1	2.38		MGI:2181647
Plin	2.37	NM_175640.1	MGI:1890505
Klk1	2.37	NM_010639.5	MGI:102850
Apod	2.36	NM_007470.3	MGI:88056
Chdh	2.35		MGI:1860776
C030044O21Rik	2.35		MGI:2444758
Lgals3	2.34	NM_010705.1	MGI:96778
Smoc1	2.32	NM_022316.1	MGI:1929878
Gys2	2.32	NM_145572.1	MGI:2385254
Tekt1	2.31	NM_011569.2	MGI:1333819
Slc6a17	2.31	NM_172271.1	MGI:2442535
Fasn	2.31	NM_007988.3	MGI:95485
Hook1	2.3		MGI:1925213
Foxq1	2.3	NM_008239.3	MGI:1298228
Ripk4	2.3	NM_023663.3	MGI:1919638
Wnt2b	2.27	NM_009520.3	MGI:1261834
Abcc8	2.27	NM_011510.3	MGI:1352629
Tjp3	2.26	NM_013769.2	MGI:1351650
Spp1	2.26	NM_009263.1	MGI:98389
4933437F05Rik	2.25	NM_027744.1	MGI:1918525
Cx3cl1	2.25	NM_009142.3	MGI:1097153
Aldh1a1	2.25	NM_013467.3	MGI:1353450
Rgs7	2.24	NM_011880.1	MGI:1346089
Stab2	2.24	NM_138673.1	MGI:2178743

1700016J18Rik	2.23		MGI:1922811
Mtap7	2.23		MGI:1328328
Crtac1	2.23	NM_145123.3	MGI:1920082
Ush1c	2.22	NM_023649.1	MGI:1919338
Kcns3	2.22	NM_173417.1	MGI:1098804
Pde4b	2.21	NM_019840.2	MGI:99557
A830073O21Rik	2.21		MGI:2443692
Gchfr	2.21	NM_177157.3	MGI:2443977
0610040J01Rik	2.2	NM_029554.2	MGI:1923511
1700007K13Rik	2.2	NM_027040.1	MGI:1916577
Zap70	2.2	NM_009539.2	MGI:99613
Zmynd10	2.18	NM_053253.2	MGI:2387863
5033411D12Rik	2.18	NM_138654.2	MGI:2178827
Klhdc7a	2.17	NM_173427.2	MGI:2444612
8430408G22Rik	2.16	NM_145980.1	MGI:1918730
Car5b	2.16	NM_019513.1	MGI:1926249
Ckmt1	2.15	NM_009897.2	MGI:99441
Tph2	2.15	NM_173391.1	MGI:2651811
Ropn1l	2.14	NM_145852.2	MGI:2182357
BC049352	2.14		MGI:3040681
Serpina1c	2.14	NM_009245.2	MGI:891969
Runx1	2.14	NM_009821.1	MGI:99852
Rprml	2.13	NM_001033212.1	MGI:2144486
Zfp697	2.13		MGI:2139736
Abca6	2.12		MGI:1923434
Gsta2	2.1		MGI:95863
Coro2a	2.1		MGI:1345966
Myog	2.1	NM_031189.1	MGI:97276
Mpzl2	2.1	NM_007962.2	MGI:1289160
AI451557	2.09	NM_001033207.1	MGI:2142553
Glis1	2.08	NM_147221.1	MGI:2386723
Klk1b5	2.08	NM_008456.2	MGI:892020
H2-Q7	2.08		MGI:95936
Ankrd32	2.08	NM_134071.2	MGI:2145448
Acaca	2.07	NM_133360.1	MGI:108451
Dock5	2.07		MGI:1916063
Fscn2	2.06	NM_172802.1	MGI:2443337
Cpne6	2.06	NM_009947.2	MGI:1334445
Cyr61	2.06	NM_010516.1	MGI:88613
Lrrc8b	2.06		MGI:2141353
Pde7a	2.05	NM_008802.1	MGI:1202402
Efhc2	2.05	NM_028916.1	MGI:1921655
Dennd2d	2.05	NM_028110.1	MGI:2181193
1190002H23Rik	2.05	NM_025427.1	MGI:1913464
Pparg	2.05	NM_011146.1	MGI:97747
Fbxl13	2.05	NM_177076.2	MGI:2443416
Ankrd5	2.04	NM_175667.2	MGI:2441685
Sncg	2.03	NM_011430.1	MGI:1298397
Il20rb	2.02	NM_001033543.1	MGI:2143266
Serpina1e	2.02	NM_009247.2	MGI:891967
LOC665918	2.02		
Spsb2	2.01	NM_013539.1	MGI:1315199
Gpnmb	2.01	NM_053110.2	MGI:1934765
Cd209f	2.01		MGI:1916392
Mbp	2	NM_001025245.1	MGI:96925
Nfkb2	2	NM_019408.2	MGI:1099800
Relb	2	NM_009046.2	MGI:103289
